# Morphometric signals of population decline in diademed sifakas occupying degraded rainforest habitat in Madagascar

**DOI:** 10.1038/s41598-019-45426-2

**Published:** 2019-06-19

**Authors:** Mitchell T. Irwin, Karen E. Samonds, Jean-Luc Raharison, Randall E. Junge, Karine Lalaina Mahefarisoa, Fidisoa Rasambainarivo, Laurie R. Godfrey, Kenneth E. Glander

**Affiliations:** 1 Department of Anthropology, Northern Illinois University, DeKalb, IL 60115 USA; 2 Department of Biological Sciences, Northern Illinois University, DeKalb, IL 60115 USA; 3 SADABE Madagascar, Antananarivo, Madagascar; 4 Columbus Zoo and Aquarium, Powell, OH 43065 USA; 5 Mahaliana Labs, Lot II B 55G Amboditsiry, Antananarivo, 101 Madagascar; 6 Department of Anthropology, University of Massachusetts Amherst, Amherst, MA 01003 USA; 7 Department of Evolutionary Anthropology, Duke University, Durham, NC 27708 USA

**Keywords:** Conservation biology, Ecophysiology, Evolutionary ecology

## Abstract

Anthropogenic habitat change can have varied impacts on primates, including both negative and positive outcomes. Even when behavioural shifts are seen, they may reflect decreased health, or simply behavioural flexibility; understanding this distinction is important for conservation efforts. This study examines habitat-related variation in adult and immature morphometrics among diademed sifakas (*Propithecus diadema*). We collected morphometric data from sifakas at Tsinjoarivo, Madagascar (19 years, 188 captures, 113 individuals). Captures spanned 12 groups, five within continuous forest (“CONT”), and seven in degraded fragments (“FRAG”) where sifakas have lower nutritional intakes. Few consistent differences were found between CONT and FRAG groups. However, using home range quality as a covariate rather than a CONT/FRAG dichotomy revealed a threshold: the two FRAG groups in the lowest-quality habitat showed low adult mass and condition (wasting), and low immature mass and length (stunting). Though less-disturbed fragments apparently provide viable habitat, we suggest the sifakas in the most challenging habitats cannot evolve fast enough to keep up with such rapid habitat change. We suggest other long-lived organisms will show similar morphometric “warning signs” (wasting in adults, stunting in immatures); selected morphometric variables can thus be useful at gauging vulnerability of populations in the face of anthropogenic change.

## Introduction

Human-induced habitat fragmentation and disturbance are causing serious declines in the extent and the viability of primate habitat, which tends to be located in the earth’s poorest nations^1^. Motivated by these declines, research investigating how primate populations and communities respond has increased. Our understanding of primate ecological responses has grown rapidly, including studies of community composition and extirpations^2,3^, diet and nutrition^4–7^, ranging and habitat use^8–10^, and activity and social behavior^11–13^. However, most of these studies have documented behavioural shifts, which may reflect underlying fitness costs (i.e., reduced survival or reproduction), or simply behavioural flexibility allowing animals to avoid such costs. It is important to avoid assuming that habitat disturbance is detrimental; low-level disturbance can increase the nutritional quality of foods, especially for folivores^14^, while frugivores typically decline in such habitats^15^.

Some studies have utilised invasive or non-invasive measures of physiologic health^16–20^, which are likely better reflections of population viability, though much remains unknown about how well these parameters predict fitness. Basic health parameters such as body size and condition are perhaps the best tools for judging population viability^21^, but with some exceptions^22–24^ these require captures, which are often difficult and risky^25^.

An individual’s body size and proportions result from a combination of genetic and non-genetic factors^26^. The genetic contribution includes adaptation to local conditions such as resource availability and predation^27^, and thus contributes to geographic variation in body size within and among species (although there may be effects of phylogenetic inertia^28^). This has been well-documented among mammals^29,30^, as well as for various lemur clades^31–35^. The non-genetic contribution encompasses many factors. First, adverse conditions during pre- and post-natal development can cause growth retardation, which may or may not be recovered through catch-up growth^36,37^, but can cause lifelong phenotypic changes in the individual and its immediate descendants via epigenetic effects^38,39^. Second, reduced resource availability for individuals, reflecting either temporal variation (e.g., lean seasons or years), spatial variation (i.e., among groups), or socially-mediated within-group differences (e.g., dominance) can impact both adults and immatures^31,40,41^, and may be reflected in body mass, circumferences and body condition indices^21,42^.

There is a large literature for humans linking adult body condition, usually expressed as body mass index (an expression of weight-per-height), to health outcomes^43^, and investigating childhood malnutrition^44^ and its links to mortality and disease^45^. However, indices like BMI are most effective within populations, since health differences and baseline shape differences among geographically separated populations can be conflated^46^. Growth is assessed by quantifying deviations from typical growth curves; growth retardation is classified into different types, specifically with “underweight” (low weight-for-age) being divided into “stunting” (low height-for-age) and “wasting” (low weight-for-height)^47^.

Few studies have investigated morphometrics in wild non-human primates with respect to habitat differences. Some have examined the effects of social conditions, such as rank (or maternal rank) on mass or growth^22,23,48^, while others have investigated seasonal changes and interannual variability^21,31^, or the effects of food provisioning, crop-raiding and foraging on human refuse^48–50^. Only six studies have directly compared habitats that are known or inferred to differ in resource availability. Mouse lemurs (*Microcebus murinus*) have reduced dry season body mass in anthropogenic secondary forest in Western Madagascar^51^, but did not vary morphometrically across three distinct habitat types in Southeast Madagascar^52^. A third study found that *M. murinus* at Ankarafantsika did not differ in mass between anthropogenic edge and interior habitats, while for a congener (*M. ravelobensis*), females, but not males, were heavier at the edge, perhaps due to higher insect prey availability^53^. *M. griseorufus* in a mesic forest were heavier and had longer skulls than in a nearby spiny forest, perhaps due to greater availability of sugar-rich fruit^54^. Finally, Japanese macaques (*Macaca fuscata*) were heavier and longer in higher-snow areas^55^, while mangabeys (*Lophocebus albigena*) at Kibale were lighter in anthropogenically disturbed (logged) compartments relative to unlogged compartments^56^. The paucity of such studies is likely due in part to the logistical difficulty of capturing a sufficiently large sample of individuals (and, ideally, groups) in each habitat being compared. All such studies of Malagasy primates have involved mouse lemurs, which live at high densities and are easily trapped; little is known about lemurs more broadly, which range from rapidly-reproducing mouse lemurs up to indri and sifakas, which have been described as “bet hedgers par excellence”^57^: female sifakas are thought to have adapted to fluctuating environmental conditions by a “slow and steady” life history and reproductive output. Specifically, sifaka females start reproducing later and continue reproducing longer for their size than other primates, which reflects a trade-off: reduced reproductive effort in any given year (thereby minimizing mortality risk in lean years) in return for a longer lifetime and more distinct reproductive opportunities.

Here, we examine morphometric data from wild diademed sifakas, *Propithecus diadema*, in relatively undisturbed continuous forest (“CONT” groups) and in disturbed forest fragments (“FRAG” groups) at Tsinjoarivo, Madagascar (Table 1), and test how body size and shape differ across this habitat gradient. Tsinjoarivo is a mid-altitude rainforest habitat atop Madagascar’s eastern escarpment; population influxes from the west have left its western portions fragmented and degraded. Sifakas present an ideal model for investigating short-term responses to habitat change, because rapid, recent habitat change^1^ has likely produced predominantly ecological rather than evolutionary responses.

**Table 1:** Group size, home range characteristics and nutritional intakes for *Propithecus diadema* study groups at Tsinjoarivo, Madagascar.

Group	Group Size^a^	Habitat^b^	Area of Botanical Plots (ha)	Basal Area/ha (m^2^/ha)	Home Range Size (ha)	Home Range Quality Index	Energy per metabolic body mass^c^ (kJ⋅BMkg^−0.762^⋅day^−1^)	Available Protein Intake^c^ (g⋅BMkg^−1^⋅day^−1^)
FRAG1	4–5	F- 24.2 ha	1.0	14.68	21.2	3.11	—	—
FRAG2	3–5	F- 44.0 ha	1.0	17.44	40.1	6.99	677	3.0
FRAG3	2–3	F- 20.9 ha	0.2	9.36	10.9	1.02	407	2.0
FRAG4	4–7	F- 228.1 ha	1.0	22.81	44.6	10.17	1084	3.3
FRAG5	2–4	F- 228.1 ha	1.0	25.50	28.0	7.14	—	—
FRAG6	3–5	F- 228.1 ha	1.0	19.85	44.1	8.75	—	—
FRAG7^d^	1–3	F- 228.1 ha	—	—	—	—	—	—
CONT1	5–10	C	1.0	39.58	83.2	32.93	1132	3.9
CONT2	3–6	C	1.0	44.71	76.0	33.97	1350	4.4
CONT3^d^	4	C	—	—	—	—	—	—
CONT4	4–9	C	0.7	35.30	90.2	31.84	—	—
CONT5	4–6	C	0.8	42.54	62.9	26.76	—	—

^a^Group size excluding infants <6 months (range of group size observed across entire study period).

^b^“C” denotes continuous forest within Madagascar’s eastern rainforest habitat while “F” denotes isolated fragment; for fragments, the total fragment size is listed.

^c^Nutritional data from Irwin *et al*.^6^ and are derived from all-day focal follows of adults and immatures quantifying feeding and intake rates, combined with food sample collection and lab analysis; ranging and habitat data from Irwin and Raharison^80^.

^d^FRAG7 and CONT3 have not been intensively studied, so data are not available for botany or ranging.

Three factors make our study system ideal for examining ecological rather than evolutionary effects of habitat change. First, groups in forest fragments have reduced nutritional inputs^6,58^ (data derived from all-day focal follows of adults and immatures quantifying feeding and intake rates, combined with food sample collection and lab analysis). Though all groups experience the lean season with reduced daily feeding time, less mass ingested, lower intakes of macronutrients, energy and minerals, and reduced activity, FRAG groups eat less and have lower nutrient and energy intakes, with the biggest divergence from CONT groups in abundant season intakes. FRAG groups (FRAG2, FRAG3, FRAG4) show higher daily feeding time than CONT groups, meaning that the difference is not due to feeding effort, but qualitative differences in the foods available^6^.

Second, fitness consequences of living in fragments is evident in the extirpation of three of seven FRAG groups. Group FRAG3 (represented in the dataset by one adult male, one adult female and one immature) was studied beginning mid-2005, and all animals died or disappeared by Sept 2006. The immature disappeared within three months of the June 2005 capture, the adult female died shortly after the July 2006 capture for no apparent reason, and the adult male died in mid-September 2006. The adult male’s remains were recovered in a tree, partially decomposed but intact and articulated; although a post-mortem exam was not possible, the body showed no sign of predation. This is consistent with a scenario in which poor nutrition contributed to death; this group had the lowest recorded nutritional inputs in the population^6^. FRAG1 is represented by just one adult female and two immatures; study began in November 2002, and all animals had died or emigrated by March 2005. This largely resulted from fosa (*Cryptoprocta ferox*) predation (as evidenced by two definitive kill sites, plus remains at a suspected kill site for a mother and infant); this unusually high predation rate appeared to be exacerbated by landscape change^59^. Finally, FRAG2 was studied between 2002 and 2014; the five individuals alive in 2014 disappeared for unknown reasons. In contrast, no CONT groups have been lost.

Third, sifakas have a very slow life history relative to the pace of habitat change. The beginning of habitat fragmentation at Tsinjoarivo gradually followed permanent local human occupation beginning in the 1980s; sifakas mature slowly (4–5 years to sexual maturity) and have life spans typically exceeding 20 years^57^. Thus, ecological changes are likely in this system while evolutionary changes due to natural selection are unlikely to have occurred. Although on one hand sifakas’ “slow and steady” life history may well make them resilient to short-term habitat variation (in terms of surviving longer than other species in lean times or lean habitats), poor conditions are still likely to be reflected in measurements of body condition, as well as rates of growth and reproduction. Additionally, living in an anthropogenically altered habitat may produce longer-term stresses, and therefore greater somatic change, compared to year-to-year changes within less-disturbed habitat. It is also worth noting that, although early socioecological theory suggested that folivorous primates were not food-limited, this has been shown to be untrue^60^, thus we do expect that habitat change has the capacity to negatively impact the food supply for (and the health of) folivores such as sifakas.

In this manuscript, we examine morphometrics of both adult and immature sifakas, considering linear measurements, body mass and several measures of condition: body condition index (BCI; a dimensionless measure of “fatness” defined as the cube root of body mass divided by body length, multiplied by 1000), as well as circumferences and testicle measurements. For adults, short-term stresses should be reflected in mass and condition, but not length, while stress during growth could cause reduced length and/or mass, which could persist into adulthood; it is therefore critical to integrate all three measures (length, mass and condition). Specifically, we asked: (1) do adult morphometrics differ between continuous/undisturbed and fragmented/disturbed habitat, and if so, which ones?; (2) are there sex-specific differences in response to disturbance (i.e., do females, who are dominant, suffer less?); and (3) which morphometrics of immatures vary among habitats? We predict that FRAG adults will have lower mass and condition (wasting) but their lengths won’t be shorter. This is because length will have been set by past conditions in a natal group, which is typically not the same group in which they were captured as adults – a CONT adult may have grown in a FRAG habitat, and vice versa. Additionally, animals growing in stressful conditions may well reach a “normal” length through a prolonged period of growth. For immatures, we predict responses in both length and mass/condition (stunting).

## Results

### Adults

Adult linear measurements did not vary among the sexes (Table 2). An effect of site was present for only one of twelve variables: trunk was longer in FRAG groups. A significant site*sex interaction was detected for body length, hind limb, big toe, ulna and hand, precluding testing of site and sex via LRTs (but the coefficients had non-significant t-values in fully loaded models). Interestingly, three of the five interactions were driven by CONT males having the lowest values, a pattern seen in seven of twelve variables. Body mass showed no effect of site or sex (Table 3), while body condition and all three circumferences were significantly higher in CONT groups. The direction of sex differences varied across habitats. For CONT individuals, females were longer in 10 of 12 linear measurements, and heavier, but had lower BCI. For FRAG individuals, males were longer in 10 of 12 measurements, and heavier, but had lower BCI.

**Table 2:** Linear measurements (mean ± SD) of adult *P. diadema* at Tsinjoarivo (n = 81 captures of 38 individuals; averages used for individuals with multiple captures; measurements in mm; differences tested using linear models).

Measurement	Overall^a^ (38)	Continuous Forest		Fragmented Forest		Effect of:		
		Females (10)	Males (8)	Females (11)	Males (8)	Site	Sex	Interaction
Tail-Crown	907.7 ± 26.4	899.4 ± 23.1	896.9 ± 27.9	909.1 ± 27.8	919.0 ± 13.2	—	—	—
Trunk	465.4 ± 15.4	463.3 ± 9.6	451.4 ± 15.7	470.8 ± 17.2	474.1 ± 9.9	F_1,35_ = 9.5, P = 0.004	—	—
Body Length	1001.3 ± 27.0	1004.6 ± 17.6	976.1 ± 30.4	1005.3 ± 26.2	1013.9 ± 22.7	—	—	F_1,33_ = 5.2, P = 0.029
Tail	441.8 ± 24.8	436.1 ± 23.9	445.5 ± 27.5	438.2 ± 25.6	442.6 ± 15.1	—	—	—
Hind limb	536.9 ± 17.4	541.3 ± 13.5	524.6 ± 17.6	534.4 ± 18.1	544.3 ± 15.7	—	—	F_1,33_ = 5.9, P = 0.020
Fibula	190.1 ± 12.0	188.8 ± 10.8	189.5 ± 15.5	192.3 ± 14.2	190.0 ± 8.4	—	—	—
Foot^b^	166.2 ± 8.6	169.6 ± 7.4	161.6 ± 13.3	166.0 ± 6.2^a^	165.8 ± 5.9	—	—	—
Big Toe	104.7 ± 7.6	106.9 ± 6.2	102.6 ± 9.7	101.3 ± 8.5	108.3 ± 3.7	—	—	F_1,33_ = 5.3, P = 0.028
Forelimb	375.8 ± 13.7	377.7 ± 14.8	368.8 ± 10.5	374.6 ± 14.1	378.1 ± 10.8	—	—	—
Ulna	149.2 ± 5.5	150.9 ± 6.5	144.2 ± 4.7	148.7 ± 3.9	151.3 ± 3.4	—	—	F_1,33_ = 8.3, P = 0.007
Hand^b^	125.2 ± 7.2	128.2 ± 7.2	123.7 ± 4.5	121.0 ± 8.6^a^	127.5 ± 5.2	—	—	F_1,33_ = 5.9, P = 0.021
Thumb	58.2 ± 4.7	59.6 ± 5.9	57.1 ± 4.8	56.9 ± 3.7	59.1 ± 4.4	—	—	—

^a^MAHA5 BP included in grand mean but not analyses; this male was translocated from CONT to FRAG habitat.

^b^Sample size = 10 rather than 11 for FRAG females (37 for grand mean).

**Table 3:** Mass, body condition index and circumferential measurements of adult *P. diadema* at Tsinjoarivo during the lean season (May-August captures only; values averaged for individuals with multiple captures; interaction term was tested but never retained; differences tested using linear models).

Measurement	Overall^a^ (34)	Continuous Forest		Fragmented Forest		Effect of:	
		Females (9)	Males (8)	Females (8)	Males (8)	Site	Sex
Body Mass, g (range)	4993 ± 338 (4200–5550)	5171 ± 302^b^ (4525–5550)	4869 ± 343 (4200–5375)	4904 ± 366^c^ (4200–5470)	4960 ± 309 (4650–5500)	—	—
Body Condition Index	17.01 ± 0.50	17.19 ± 0.36	17.40 ± 0.52	16.75 ± 0.36	16.69 ± 0.48	F_1,31_ = 14.5, P = 0.001	—
Chest Circumference, mm	295.8 ± 14.6	301.0 ± 10.0	303.1 ± 17.9	285.7 ± 11.2	291.4 ± 14.2	F_1,31_ = 8.4, P = 0.007	—
Biceps Circumference, mm	108.4 ± 5.4	110.2 ± 4.5	111.6 ± 6.3	103.4 ± 4.0	107.7 ± 3.6	F_1,31_ = 9.8, P = 0.004	—
Thigh Circumference, mm	178.9 ± 11.3	184.6 ± 11.7	184.9 ± 9.0	171.3 ± 5.9	173.3 ± 11.5	F_1,31_ = 14.0, P = 0.001	—

^a^MAHA5 BP included in grand mean but not analyses; this male was translocated from CONT to FRAG habitat; includes captures of pregnant females who might be expected to affect results for mass and BCI, but re-running the analysis with pregnant captures excluded does not change test results: neither factor is significant for mass, and site is retained as the sole factor retained in the model predicting BCI (F_1,31_ = 13.4, P = 0.001).

^b^Removing values for captures of pregnant CONT females yields slightly lower mean (5155 ± 319 g, n = 6).

^c^Removing values for captures of pregnant FRAG females yields slightly lower mean (4855 ± 330 g, n = 8).

Adult males’ testicle measurements were higher in rainy season captures. In the dry season, testicle length did not differ among habitats, but testicle width and volume were significantly lower in FRAG groups, as was the ratio of testicle volume to body mass (Table 4). Rainy season testicle measurements showed similar trends but small sample size precluded statistical testing.

**Table 4:** Testicular measurements of *P. diadema* at Tsinjoarivo (using individual averages for animals with multiple captures) during dry season (May-August) and early rainy season (Oct-Nov); differences tested using Wilcoxon rank sum tests.

	Both Sites^a^	Continuous Forest	Fragmented Forest	Effect of Site:
May-August Testicle Length (mm)	18.6 ± 1.6 (17)	18.7 ± 1.8 (8)	18.4 ± 1.5 (8)	W = 35, *P* = 0.8
May-August Testicle Width (mm)	11.7 ± 1.6 (17)	12.7 ± 1.3 (8)	10.7 ± 1.4 (8)	W = 54, *P* = 0.024
May-August Testicle Volume (mm^3^)	2732 ± 860 (17)	3212 ± 861 (8)	2235 ± 632 (8)	W = 52, *P* = 0.038
May-August TV:BM Ratio	0.55 ± 0.17 (17)	0.66 ± 0.15 (8)	0.45 ± 0.12 (8)	W = 57, *P* = 0.007
Oct-Nov Testicle Length (mm)	26.3 ± 2.2 (4)	25.6, 29.1 (2)	23.7, 26.7 (2)	n/a
Oct-Nov Testicle Width (mm)	17.6 ± 2.0 (4)	16.5, 19.9 (2)	15.5, 18.7 (2)	n/a
Oct-Nov Testicle Volume (mm^3^)	8750 ± 2680 (4)	7255, 12005 (2)	5977, 9762 (2)	n/a
Oct-Nov TV:BM Ratio	1.67 ± 0.43 (4)	1.34, 2.11 (2)	1.26, 1.95 (2)	n/a

^a^MAHA5 BP included in grand mean but not analyses; this male was translocated from CONT to FRAG habitat.

**Figure 1:**
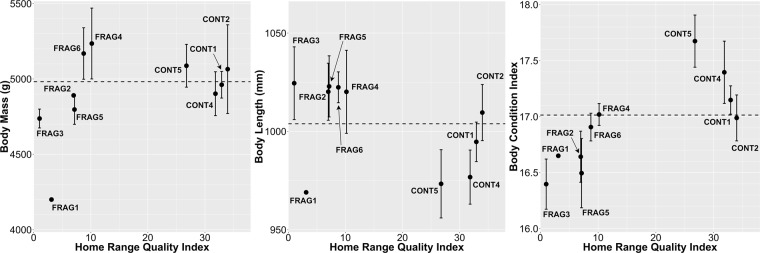
Adult body mass, body length and body condition index as a function of home range quality index for *Propithecus diadema* at Tsinjoarivo, Madagascar. Data from lean season captures only and using individual averages for repeatedly captured individuals; plotted points show mean ± SE; dashed horizontal lines indicate sample-wide means for lean season captures (4982 g mass, 1004 mm body length,17.014 BCI), sample size: CONT1: 5, CONT2: 4, CONT4: 4, CONT5: 3, FRAG1: 1 FRAG2: 2, FRAG3: 2, FRAG4: 2, FRAG5: 3, FRAG6: 3.

When HRQI was used to contextualize adult morphometrics, rather than a CONT/FRAG dichotomy, a relationship becomes apparent (Fig. 1). Among FRAG groups, those with higher HRQI (i.e., FRAG4, FRAG6) showed average or above-average mass, length and BCI relative to CONT groups. Those with the lowest HRQI (i.e., FRAG1, FRAG2, FRAG3, FRAG5) were more variable. Two FRAG3 adults showed high body length but low mass and BCI, while the one adult from FRAG1 (a 5-year-old natal female) was low in all three measures. Group-level Spearman’s rank-correlation tests (n = 10) showed a significant positive association between BCI and HRQI, (rho = 0.78, *P* = 0.012), which seems to be driven by low mass at low HRQI (positive relationship between mass and HRQI; rho = 0.61, *P* = 0.066) and higher average body length at low HRQI (rho = −0.41, *P* = 0.24). Particularly for body mass, there seems to be a non-linear relationship, with reduced mass in groups below a threshold at HRQI = 7 but little variation in better habitats.

### Immatures

**Figure 2:**
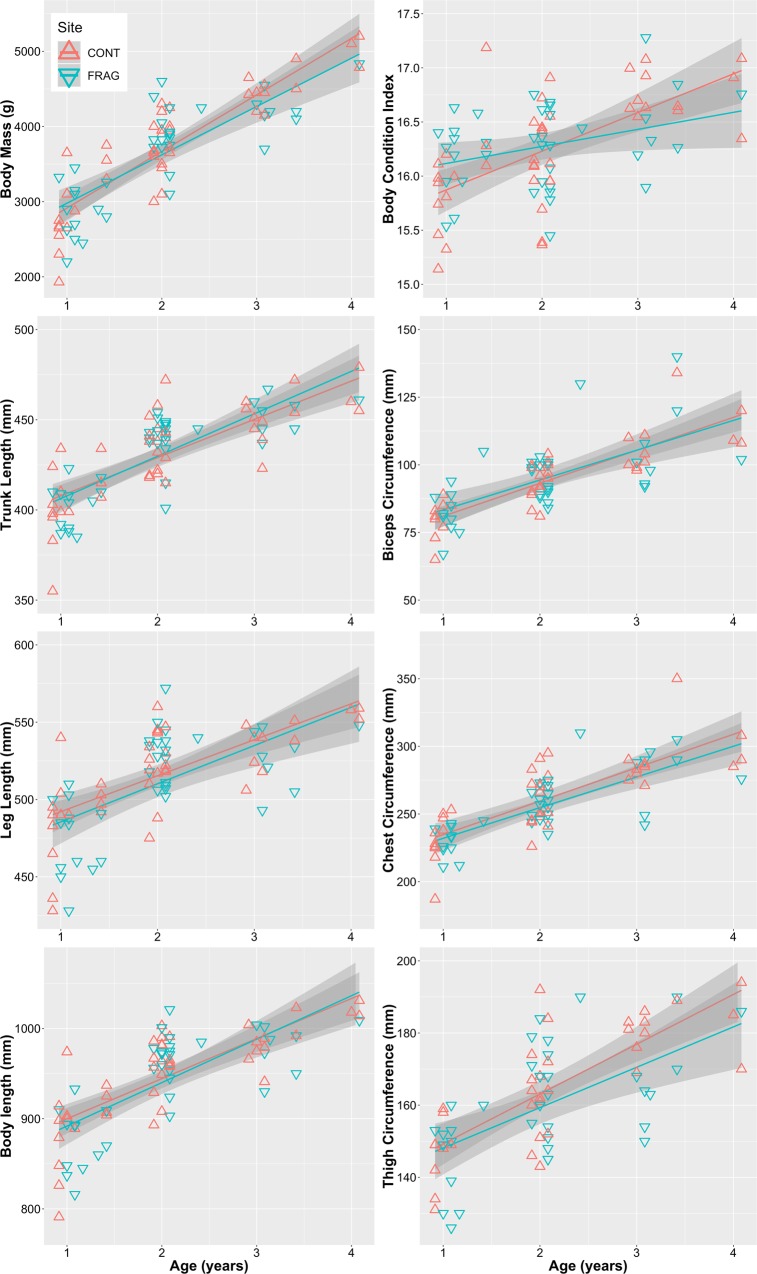
Growth of *Propithecus diadema* immatures in continuous and fragmented forest at Tsinjoarivo for eight morphometric variables, with site-specific linear models and 95% confidence intervals.

**Figure 3:**
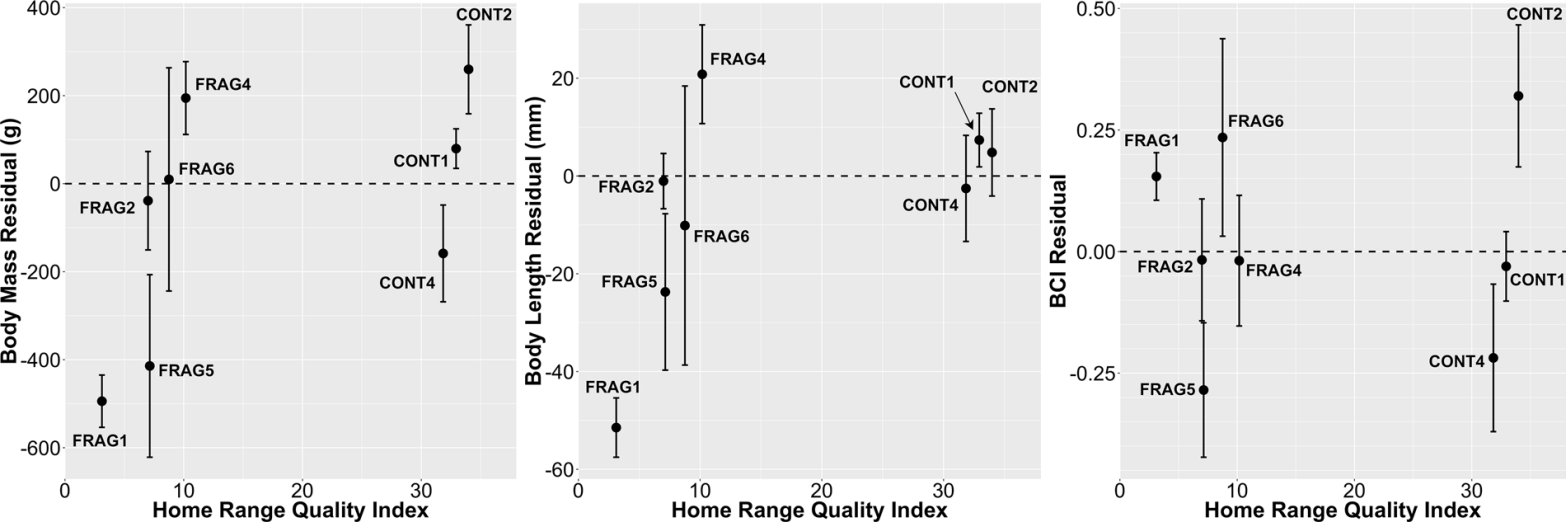
Immatures’ body mass residual (body mass at capture minus body mass predicted from age), body length residual and BCI residual as a function of home range quality index for *Propithecus diadema* at Tsinjoarivo, Madagascar. Data from all seasons and using individual averages for repeatedly captured individuals; plotted points show mean ± SE; dashed horizontal lines indicate a zero residual (i.e. “typical” value for age across the sample); sample size: CONT1: 15–16, CONT2: 7, CONT4: 11, FRAG1: 2, FRAG2: 6, FRAG4: 10, FRAG5: 4, FRAG6: 4.

Measurements of immature sifakas tended to increase linearly with age during the examined age range (Fig. 2). Age was (as expected) a significant positive predictor for all measurements, but site was not retained in any of the models (Table 5), and no age*site interaction terms were significant. When HRQI was used to contextualize immature morphometrics rather than a CONT/FRAG dichotomy, a relationship emerged (Fig. 3). HRQI was significantly positively correlated with mass (Spearman’s rho = 0.76, *P* = 0.037, n = 8); groups with low HRQI tend to have shorter length (rho = 0.67, *P* = 0.08), while the relationship between BCI and HRQI is flat (rho = 0.05, *P* = 0.9). For mass and body length, the response seems non-linear, with reduced values below a threshold of roughly HRQI = 7 (individuals in groups FRAG1 and FRAG5 in particular displayed lower-than-expected values). FRAG2 performs better than expected considering its low HRQI.

**Table 5:** Linear Mixed Models describing growth parameters as a function of age and habitat for *Propithecus diadema* at Tsinjoarivo aged 0.5–4.5 years; n = 81 captures of 62 individuals in 10 groups except for body mass and BCI (80 captures, 61 individuals, 10 groups) and circumferences (74 captures, 55 individuals, 9 groups); predictor variables tested using linear mixed models.

Variable^a^	Age	Site	Interaction
Body Mass (g)	**β** = **+730, LR** = **119.5, P** < **0.0001**	ns - LR = 0.87, P = 0.35 (β = −139.2)	ns
Trunk Length (mm)	**β** = +**22.1, LR** = **75.4, P** < **0.0001**	ns - LR = 0.07, P = 0.8 (β = + 0.90)	ns
Hind limb Length (mm)	**β** = +**26.0, LR** = **54.9, P** < **0.0001**	ns - LR = 1.24, P = 0.27 (β = −9.03)	ns
Body Length (mm)	**β** = +**47.5, LR** = **69.9, P** < **0.0001**	ns - LR = 0.42, P = 0.5 (β = −6.47)	ns
BCI	**β** = +**0.27, LR** = **37.6, P** < **0.0001**	ns - LR = 0.003, P = 1.0 (β = + 0.01)	ns
Biceps Circumference (mm)	**β** = +**10.41, LR** = **64.4, P** < **0.0001**	ns - LR = 0.13, P = 0.7 (β = + 1.61)	ns
Chest Circumference (mm)	**β** = +**23.5, LR** = **67.1, P** < **0.0001**	ns - LR = 1.19, P = 0.28 (β = −4.76)	ns
Thigh Circumference (mm)	**β** = +**13.1, LR** = **50.6, P** < **0.0001**	ns - LR = 1.25, P = 0.26 (β = −4.32)	ns

^a^Coefficients reported as effect of living in FRAG group relative to CONT group, or the effect of one year of growth; when effect of site was not retained in the model, the coefficient (beta weight) of the site effect was reported for the model including both fixed factors, for illustrative purposes only.

Body masses of 17 immatures less than 6 months old are presented in Supplementary Table S1. There are too few data to test for habitat differences, but typical neonate mass appears to be approximately 140 g.

## Discussion

This study revealed habitat differences in some, but not all, morphometric variables, as well as key differences between adults and immatures in the differences observed. It also revealed that the altered morphometric signals were not universal to all FRAG groups, but tended to be restricted to those in the lowest-quality home ranges.

Adult sifakas at Tsinjoarivo showed body mass and shape variation across habitats but only in some variables. We expected that FRAG adults would not be shorter than CONT adults, and this was true. Only one linear variable differed significantly among habitats: CONT animals had shorter trunks. As expected, FRAG individuals tended to be lighter, but not significantly so; however, all four body shape variables (BCI plus three circumferences) were significantly lower in FRAG groups. These results are consistent with wasting in FRAG groups, but the signal appears complicated: FRAG adults have similar mass to CONT adults but distribute that mass over a longer frame (particularly a longer trunk). The signal is somewhat clearer when using HRQI as a covariate rather than a CONT-FRAG dichotomy: this approach suggests a threshold effect. Wasting is seen only in FRAG adults occupying the two poorest-quality home ranges, FRAG1 and FRAG3 (significant positive BCI-HRQI correlation, near significant mass-HRQI correlation, and no correlation between length and HRQI). Testicle volume is higher in the rainy season (when mating occurs), mirroring changes seen in *P. edwardsi*^61^ and *P. verreauxi*^62^. In the dry season (when birth occurs), FRAG males apparently invest less energy in testis tissue and allow testes to shrink more than CONT males. Since this variation can’t be explained by intragroup competition (all groups were single-male), there are two possible explanations. First, nutritional stress in FRAG groups may cause more tissue loss. Second, some FRAG males live in isolated fragments, and thus may perceive a low risk of male invasion (e.g., fewer extragroup scentmarks), and invest less in testes. We favour the first interpretation, as the average testis volume in the three isolated groups (2480 mm^3^) was higher than in the four non-isolated groups (2153 mm^3^).

We also predicted that sexes would respond to habitat differently. Females tended to be heavier, similar to *P. edwardsi* at Ranomafana^42^. This contributes to the mixed evidence for sexual dimorphism within the genus: *P. verreauxi* at Kirindy, *P. tattersalli* at Daraina and *P. coquereli* at Ankijabe are dimorphic, but *P. verreauxi* are monomorphic at Bevola, and at Beza Mahafaly except during late dry season when males are heavier^21,63,64^. Three variables showed a sex-by-site interaction. Females are longer and heavier with lower BCI in CONT groups, but the opposite is true in FRAG groups. Superficially, this suggests that the sexes experience resource stress differently, with female dominance more strongly expressed in better habitat (possibly because resources are more contestable). However, the fact that the main driver of this effect is unusually short CONT males (who experience high HRQI and nutrient intakes) defies simple explanation.

Immatures showed a similar threshold effect: FRAG immatures were not consistently affected, but those in the worst groups showed stunting, as predicted: low mass and length, but “normal” BCI. The increase in BCI across ages 1–4 corroborates previous research in a congener, *P. edwardsi*^42^. These researchers found that linear growth is more accelerated (animals reach adult length at ~2 years) than growth in mass (animals reach adult mass at ~6 years); in other words, animals lengthen, and then fill out. However, Tsinjoarivo sifakas appear to reach adult length later (~3–4 years), suggesting that resource availability may be lower than at Ranomafana.

To interpret this variation, one must consider the different contexts of the adult and immature datasets. Immature morphometrics should simply reflect conditions in the current home range, while adult morphometrics should reflect factors that acted during growth (probably more influential in fixed variables such as skeletal length) as well as conditions in the months preceding capture (probably more influential in plastic measures like circumferences and BCI). Thus, a full understanding of adult variation should consider both the natal home range and the current home range; for most adults we know the latter but not the former. FRAG adults probably grew up in poorer habitats than CONT adults on average, but the maximum distance among groups is just 12 km, possibly within their dispersal capabilities. Further, FRAG adults might have matured before habitat change was fully manifested. The fact that adults’ “fatness” (BCI and circumferences) and testicle volume varied among sites more than linear variables confirms that length may be determined more by genetics and/or natal group conditions, and “fatness” more by current conditions.

Broadly, there are four possible explanations for variation across habitats such as we observed. First, differences may reflect genetically-coded adaptations to local habitat. However, as previously explained, we consider an evolutionary explanation unlikely, since disturbance began as recently as 35 years ago (~2 sifaka generations), and gene flow across the landscape was probably continuous until even more recently.

Second, speed and patterns of growth may vary due to nutrition. This seems likely here: reduced nutritional inputs in three of the FRAG groups were previously documented^6,65^; these groups varied considerably in energy and macronutrient inputs, and the ordering of those inputs (FRAG3 < FRAG2 < FRAG4) matches their ordering in HRQI, adult mass and BCI (but not length). Adults likely arrived in their current groups with skeletal dimensions set by genetics and/or natal habitat, and local conditions then determined their “fatness” (wasting). The contrasting signal in immatures (low mass and length, but not BCI, in the most degraded home ranges) is consistent with retarded growth in both mass and length (stunting).

One key test of this hypothesis would be observations of the same individual before and after natal dispersal. We don’t have the data to test this definitively, but one instructive example is the adult male Black-Gold, who transferred from group FRAG1 (HRQI = 3.11) to FRAG4 (HRQI = 10.17). The comparison is difficult because his captures were in different seasons, but he did increase in mass and condition between capture in FRAG1 (early rainy season; mass = 4750 g, BCI = 16.79), and FRAG4 (dry season; mass = 5000 g, BCI = 17.11).

The shorter bodies of CONT males were unexpected under a “nutritional constraints” scenario. Unfortunately, there is almost no contextual literature for primates: in the only studies of adult primates’ mass and proportions across richer and poorer habitats, Olupot^56^ found that male mangabeys were lighter and had a lower mass:length ratio in logged areas, as predicted. Although there is evidence that nutrition during growth affects humans’ body proportions in ways beyond simple stunting and wasting^66,67^, further research is necessary to explain this result.

Third, endocrinological differences between habitats, in both intrauterine exposure and endogenous production during growth, may affect growth. Androgens can affect bone modelling, longitudinal growth and epiphyseal closure^68^, and have been linked to higher infant growth velocities and earlier maturity in adolescents^69,70^. Lemurs might be somewhat unique in endocrine exposure during growth; Kappeler and Fichtel^71^ proposed a model explaining lemurs’ unique social and physiological characteristics via a canalization effect, with high competition amongst adult females causing high prenatal androgen/estrogen ratios and masculinization of daughters as a by-product. FRAG mothers might experience higher stress, causing higher-androgen intrauterine environments; this could explain the longer, skinnier males seen in FRAG groups.

Finally, we also must consider the possibility that adult individuals in each habitat (CONT and FRAG) may be more closely related to one another and therefore not entirely independent samples. If a large proportion of adults in one habitat were all descended from a recent ancestor, this could cause a statistically significant but spurious difference among habitats. Unfortunately, we currently know too little about sifaka intergroup dispersal to be able to judge the likelihood of this effect.

One complicating factor in interpreting our results is interannual variation. Madagascar’s climatic and environmental variation is considerable^72^, and this may have shaped lemurs’ physiological and ecological adaptations^57,71,73^. Unlike some lemur populations, Tsinjoarivo’s inland biogeographic position largely buffers it from cyclones, and its position in the rainforest protects it from droughts (the lowest recorded annual rainfall was 1613 mm). However, Madagascar shows considerable interannual variation in fruiting, which seems to be stronger in rainforests; for example, Ganzhorn *et al*.^74^ found that western deciduous forest trees fruited with a probability of 72% per year, while lemur food trees in eastern rainforest had only a 40% probability. Sifaka diet at Tsinjoarivo varies considerably from year to year, with many key rainy season fruits having supra-annual fruiting schedules (Irwin, unpub. data); nutritional inputs may be similarly variable. Our morphometric data show considerable year-to-year variation, and this variation is more extreme for mass than for length. For example, the mass of individuals captured around 2 years of age (23–25 months) varied from 3100 to 4600 g (mean = 3838, SD = 346, CV = 9.0) while body length varied from 903 to 1021 mm (mean = 966, SD = 26, CV = 2.7). This is consistent with research on Milne-Edwards’ sifakas (*Propithecus edwardsi*), which found considerable year-to-year morphometric variation within individuals^42^ and variation in reproductive success linked to year-to-year climatic variation^75^.

We do not have the data to explore this link fully; future studies should compile phenology and nutritional sampling across multiple years to reveal the degree of variation^76^, and the degree to which this variation is linked to climate. More broadly, global and/or local climatic variables may be simple, useful correlates of habitat productivity in a given year^75^, which could be incorporated into models as covariates; however, these data may not correlate well with local food availability. Finally, indirect measures of stress such as faecal glucocorticoids and metabolites in faeces and urine could be more accessible proxies for the richness of a given year^18,77^; a correlation between a proxy for stress and morphometric variables in a given year would support nutritional stress as a mechanism affecting morphometric outcomes. Future efforts to quantify interannual variation in resource availability and nutritional inputs will be an important part of understanding how Madagascar’s variable environment impacts fitness outcomes.

Altogether, our results revealed an important habitat threshold. Some FRAG groups, despite occupying smaller and more disturbed home ranges than CONT groups, did not have poorer growth outcomes; negative impacts appear only at HRQI below a threshold of roughly 7. It should be noted that one of the weaknesses of our dataset is an under-representation of those groups, mainly due to factors outside our control. Continued sampling of these groups would have been a priority, but was not possible. At the beginning of the study, we deliberately targeted groups in the smallest fragments; when groups were lost, we replaced them with groups in the smallest fragments still occupied. However, although anecdotal, the loss of these three groups (and the absence of outside forces causing death for FRAG3) corroborates the interpretation that their health was impacted by their low-quality habitat. Had they persisted, they would have likely been demographic sinks in a larger metapopulation; now that they are extinct it is unclear whether these habitats will be recolonized. For the remaining FRAG groups, both nutritional inputs^6^ and morphometric data show little distinction from CONT groups, despite a noticeable degree of habitat fragmentation and disturbance (e.g., FRAG4’s HRQI is 10.3 – one-third of CONT groups). This suggests substantial resilience; if no further degradation occurs, the remaining FRAG groups’ long-term viability may depend more on dispersal capabilities than on nutritional inputs. If natal dispersal is limited because individuals are unwilling to traverse long distances between fragments, or due to high mortality during dispersal, reduced recruitment into the adult population and/or inbreeding may result.

To date, few studies have demonstrated links between habitat fragmentation/degradation and morphometrics in primates, yet an improved understanding of differences in body mass and size between disturbed and undisturbed habitat would be useful in understanding threats, setting conservation priorities and identifying populations at risk. Further, these data would shed light on the interplay between evolutionary and ecological forces in determining an individual’s size and shape, both during growth and in adulthood. Our findings reveal some clear effects of habitat change on sifakas in line with predictions, yet other elements of our dataset are puzzling and allow only speculation regarding root causes. Continuing study is needed to better understand this complex picture, and will be especially important for long-lived species like sifakas, who may persist for many years in sink habitats due to dispersal from source habitats, yet may not actually constitute viable populations in the long run.

## Methods

### Study site

Tsinjoarivo Forest is located 80 km SSE of Antananarivo, atop the escarpment dividing Madagascar’s central plateau from the eastern lowlands. This region contains unprotected, central domain mid-altitude rainforest, within a previously-continuous corridor including Ranomafana (150 km SSW) and Andasibe-Mantadia (100 km NE) National Parks. The corridor’s western half has been fragmented and degraded by settlers from the central plateau, while the eastern half is minimally disturbed^78^.

We established three camps at Tsinjoarivo. Ankadivory (19°42′59′′S, 47°49′18′′E, 1345 m) and Vatateza (19°43′15″S, 47°51′25″E, 1396 m) are within continuous forest in the central/eastern part of the forest corridor; both have some human settlement, but limited human disturbance within the forest. Mahatsinjo (19°40′56″S, 47°45′28″E, 1590 m), approximately 12 km to the northwest, is within a network of hill- and ridge-top forest fragments. Mahatsinjo is roughly 50% forested, and fragments show altered structure (reduced tree density, crown volume, and basal area per hectare) and lower tree diversity relative to Vatateza^78^. Here, Ankadivory and Vatateza are referred to as continuous forest (“CONT”) sites and Mahatsinjo is referred to as fragmented forest (“FRAG”). Sifakas are not currently hunted within the study areas; local residents relate that sifakas experienced low-level blowgun hunting in the past, but this has not been observed in the study area since 2000.

Rainfall at Vatateza totals 2632 mm (average, 4.5 years), of which 1697 mm (64%) falls during December-March. Rainfall at Mahatsinjo is lower, 2083 mm (average, 15 years), with 1307 mm (63%) falling during December-March. Temperature is highest in December-January (max/min: 26.1/13.8 °C) and lowest in June-August (max/min: 17.9/7.0 °C). We refer to the hotter, rainy season (November to mid-April) as the “abundant season” because of higher availability of flowers, fruit and young leaves^8^ compared to the “lean season” (mid-April through October).

### Study subjects and capture methods

Diademed sifakas (*Propithecus diadema*) are reported to be both the largest *Propithecus* species, and the largest extant lemur, at roughly 6.5 kg^35^, and resemble other *Propithecus* and many of the gregarious lemurs in being female-dominant^73^. They have dental and digestive adaptations to folivory, and foliage is their top food, but occupies only 42–53% of feeding time^4,79^; they apparently prefer fruit or seeds, consuming these in large quantities when available.

Tsinjoarivo sifakas live in small groups (2–10 individuals, excluding infants) containing 1 adult male, 1–2 adult females, and up to 7 immatures. Typically, offspring disperse from natal groups beginning at 5 years, and subsequently breed, but two females reproduced in their natal group (birth at 4 years; conception at 3.5 years, i.e., before cessation of growth; Irwin, unpub. data). Breeding is strictly seasonal, with mating in December, birth in June-July and weaning in roughly January-February (Irwin, unpublished data).

**Figure 4:**
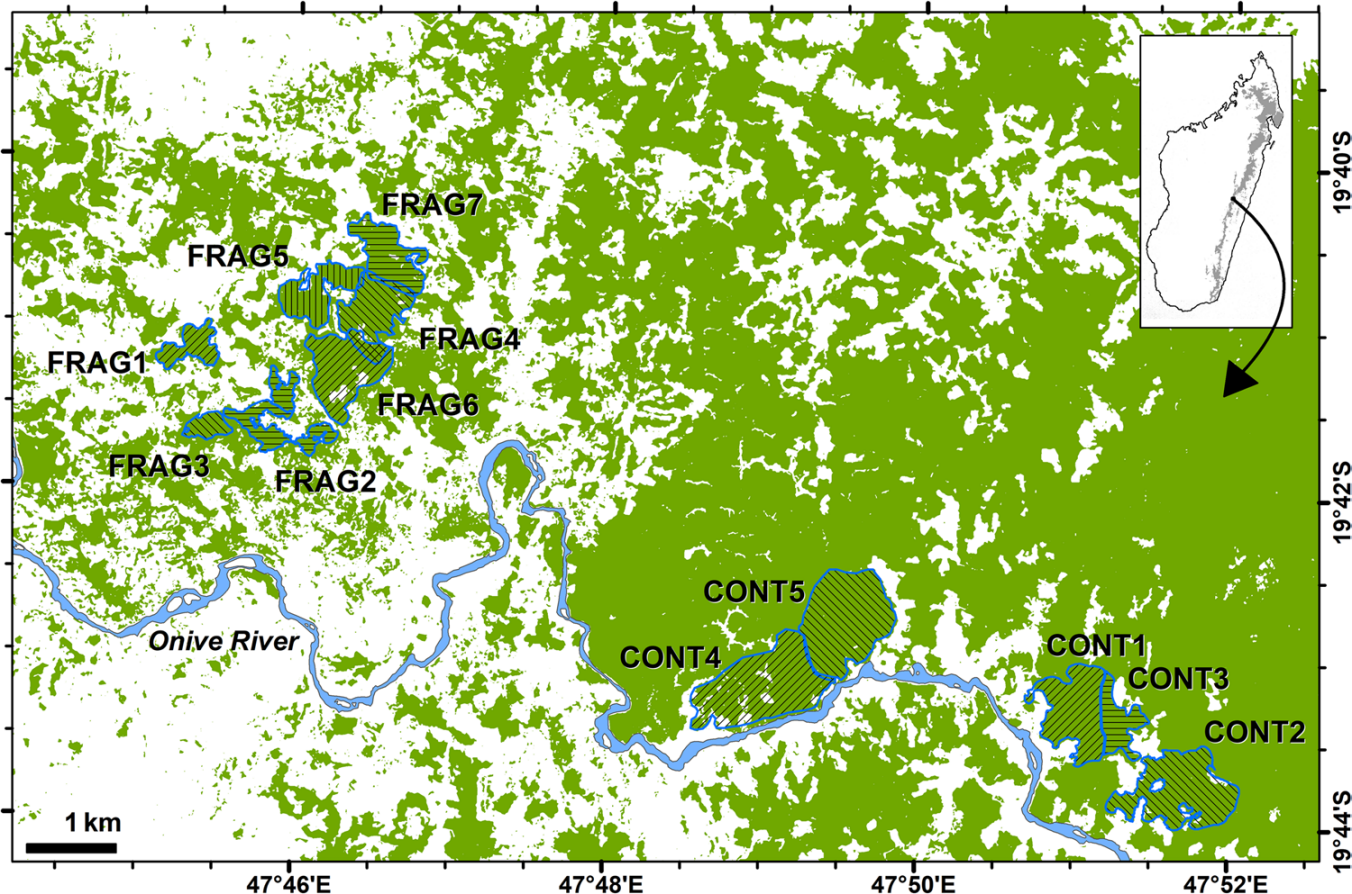
Location of *Propithecus diadema* study groups at Tsinjoarivo; green shows forest cover and hatched areas indicate approximate home range locations.

Twelve groups were examined: five at Vatateza (CONT1, CONT2, CONT3) and Ankadivory (CONT4, CONT5) and seven at Mahatsinjo (FRAG1-7; Fig. 4; Table 1). Four FRAG groups share one larger fragment, while the other three occupy (or occupied) isolated fragments. Three early captures preceded the establishment of study groups. As dichotomous habitat classification (CONT vs. FRAG) masks underlying continuous variation in habitat quality, we calculated “home range quality index” (HRQI), defined as the home range (ha) multiplied by “basal area per hectare” (cumulative basal area of trees ≥5 cm DBH; measured as m^2^/ha; Table 1). Basal area per hectare roughly correlates negatively to the degree of past timber extraction at Tsinjoarivo, with lower values in FRAG groups^80^. DBH and basal area are positively correlated with both leaf and fruit biomass for individual trees and at the landscape level, and have been used to explore population densities^3^; however, in examining quality of a specific home range, it is more appropriate to multiply this measure by home range area^81^, so that both resource density and overall area are considered. Variation in HRQI may reflect fruit production more than leaf production (and therefore be more relevant for frugivores), but we felt this was justified since sifakas apparently prefer fruit, consuming it in relation to its availability, and fruit delivers higher energy intakes^4,6^. HRQI ranged from 1.02 (poorest) to 33.97 (richest). Finally, botanical plots and phenological data from 2003 are consistent with the link between basal area and resource production: CONT habitat (CONT1 and CONT2) had roughly double the crown volume per hectare (trees >5 cm DBH) compared to FRAG groups (FRAG1, FRAG2) and per-hectare resource availability scores (phenology scores weighted by crown volume) were higher in CONT habitat for both fruits and leaves^78^. Group-specific nutritional intakes scale with HRQI (Table 1): FRAG4 (HRQI = 10.17) showed a 13% reduction in energy consumption and a 20% reduction in protein consumption relative to CONT groups; FRAG2 (HRQI = 6.99) showed 45% and 28% reductions, while FRAG3 (HRQI = 1.02) showed 67% and 52% reductions.

As part of a long-term study, we performed 188 captures between October 1999 and June 2018. Captures were performed with Pneu-darts™, loaded with Telazol® at 25–30 mg/kg body mass, and projected by a CO_2_-powered projector or blowgun^82^. These included 81 captures of 38 adult individuals (≥5 years old) and 107 captures of 84 immatures (0–4.1 years old); of the immatures, 90 were captured independently (0.9–4.1 years old) and 17 were captured as dependent infants during their mother’s capture. We mitigated the risk of capturing females with infants by increasing personnel, reducing darting distance, darting only when infant position was seen and fully obscured by mother’s body, and darting only in low vegetation. Infants were not anaesthetized and were weighed but not measured. Nine individuals were in both databases (captured as immatures and later as adults).

Body mass was measured using Pesola spring scales (300 g, 1 kg, 5 kg, or 10 kg). Linear somatic measurements (Supplementary Table S2) were made using a rigid metal tape measure, testicular measurements using dial calipers, and circumferential measurements using a pliable tape measure. Measurements for the first three captures were performed by KEG (1999), and the following 20 (2002–2003) were performed by KEG, MTI, KES, and JLR (during which KEG trained the others); for remaining captures MTI, KES and JLR performed measurements. Using these measurements, we calculated:

1 $${\rm{Body}}\,{\rm{length}}={\rm{tail}} \mbox{-} {\rm{crown}}\,-\,{\rm{tail}}\,{\rm{length}}+{\rm{hind}}\,{\rm{limb}}$$

2 $${\rm{Trunk}}={\rm{tail}} \mbox{-} {\rm{crown}}-{\rm{tail}}\,{\rm{length}}$$

3 $${\rm{Body}}\,{\rm{Condition}}\,{\rm{Index}}\,({\rm{BCI}})=\frac{{(bodymass)}^{1/3}}{body\,length}\times 1000$$

4 $${\rm{Testicle}}\,{\rm{volume}}=4/3\pi (0.5\cdot {length})\,{(0.5\cdot {width})}^{2}$$

We calculated the correlation structure for morphometric variables and determined that the degree of redundancy is low, particularly for body mass vs. most lengths. We present all measurements because these are the first published wild morphometric data for this species and because little is known about how strongly correlated the measurements are, and how allometry^83^ or stress during growth (nutritional or mechanical) affects body segments differentially. For example, variation in hand and foot proportions may correlate with structural differences between CONT and FRAG habitats.

### Data analysis

Our captures cluster in two seasons: May-August (163) and October/November (25). Due to seasonal diet differences^6^, we expect mass, circumferences and testicle dimensions to fluctuate seasonally^42^, thus different analyses employed different databases. All statistical analyses used R^84^.

For adult linear measurements, we used individual averages from all captures (81 captures, 38 individuals); for adult mass, condition and circumferences we used individual averages only from May-August captures (70 captures, 34 individuals). We used linear models (function “lm”) to assess the effects of site (CONT vs. FRAG), sex and site*sex interaction. Group was not included as a random effect since most sifakas disperse from natal groups^85^; thus adult group mates have distinct life histories. However, because group mates may converge in some more plastic variables over time (mass, circumferences, body condition) those variables were tested using linear mixed models (using group as a random effect); results did not differ from the linear models presented here. Model fitting followed three steps and used likelihood ratio tests (LRTs; threshold for inclusion *P* < 0.05). First, a fully loaded model (two fixed effects, one interaction) was compared to a model with no interaction term; the simplified model was adopted if the interaction did not significantly improve model fit. If the interaction was retained, fixed factors were not tested; otherwise, sex and then site were tested (in that order) and retained if the LRT was significant. For testicles we present data from each season separately, again using individual averages for animals with multiple captures. We tested for an effect of site (for May-August data) using a Wilcoxon rank sum test. We excluded one adult male (FRAG5:BP) from testing because he was translocated from CONT to FRAG habitat, and his morphometrics therefore reflect a mixture of CONT conditions during growth and FRAG conditions at capture. For analyses that included pregnant individuals, we double-checked results against a database with pregnant individuals excluded.

To examine morphometrics of immatures, we trimmed the dataset to exclude 17 individuals <0.5 years old (because they may be at least partially buffered from resource challenges through lactation), and 9 individuals with unknown age, yielding 81 captures of 62 individuals. Among these, 71 captures had age known (to the nearest month) through observation of birth/infant stages; for the remaining 10 we had reliable age estimates derived from independent physical evidence (e.g., minimally-erupted canines are seen only before ~1.5 years) or constraining demographic information. We used linear mixed models (LMMs) to assess the effects of site, age and site*age interaction on morphometric variables. We did not transform age because data exploration revealed roughly linear increases with age over this age range. Because groups and individuals were sampled repeatedly, both group (n = 10) and individual nested within group (n = 62) were included as random effects (intercept only) in the model. Model fitting followed three steps and used LRTs (using ML model fitting; threshold for inclusion *P* < 0.05). First, a fully loaded model (two fixed effects with interaction, two nested random effects) was compared to a model with no interaction; the simplified model was adopted if the interaction did not significantly improve model fit. If the interaction was retained, fixed factors were not tested individually; otherwise, age and then site were tested (in that order) and retained in the model only if the LRT was significant. Final model results are reported using restricted maximum likelihood (REML) models. We did not include sex because sifakas are sexually monomorphic; for a re-analysis (not shown) including sex, this factor was always dropped and models reduced to those presented here. LMMs used the nlme package^86^.

For adults, we plotted mass, body length and BCI against HRQI, while for immatures we plotted residuals for mass, body length and BCI (derived from a simple linear model predicting mass based on age from the sample of 81 captures, with no random effects). This analysis included only 29 adults and 59–60 immatures as some groups lacked botanical data and HRQI. Because morphometrics may vary with HRQI in a non-linear fashion, we used Spearman’s rank-correlation tests to examine correlations between morphometrics and HRQI at the group level.

Finally, we report body mass for individuals less than 6 months old. For one of these, age at capture was known exactly due to observation of birth; for others birthdate was constrained by observations before and after birth, and using inferences from developmental stage and behaviour, as compared to known-aged infants. We did not compare CONT and FRAG infant weights because of small sample size, and the variation among individuals in age at capture.

### Ethical approval

This research complied with protocols approved by Stony Brook University and Northern Illinois University IACUC, McGill University’s Animal Care Committee, and University of Queensland’s Animal Ethics Committee, and was carried out in accordance with the legal requirements of Madagascar, the Code of Best Practices for Field Primatology (American Society of Primatologists), and the Guidelines for the Capture, Handling and Care of Mammals (American Society of Mammalogists); research permits were issued by Madagascar’s Ministry of Ecology, Environment, and Forests (most recent: #106/18/MEEF/SG/DGF/DASP/SCB.Re).

## Supplementary information

Supplementary Information

## Data Availability

The datasets generated during and/or analysed during the current study are available from the corresponding author on reasonable request.
