# Supplementary material for: Morphometric signals of population decline in diademed sifakas occupying degraded rainforest habitat in Madagascar
Source: Sci Rep. 2019 Jun 19;9:8776. doi: 10.1038/s41598-019-45426-2 (PMC6584568; doi:10.1038/s41598-019-45426-2)
Supplement: Supplementary file 1 — Supplementary Information [file 41598_2019_45426_MOESM1_ESM.docx]

**Morphometric signals of population decline in diademed sifakas occupying degraded rainforest habitat in Madagascar**

**Supplementary Information**

# Mitchell T. Irwin^1^*

Karen E. Samonds^2^

Jean-Luc Raharison^3^

Randall E. Junge^4^

Karine Lalaina Mahefarisoa^3^

Fidisoa Rasambainarivo^5^

Laurie R. Godfrey^6^

Kenneth E. Glander^7^

^1^ Department of Anthropology, Northern Illinois University, DeKalb, IL, USA 60115

E-mail: mirwin@niu.edu

^2^ Department of Biological Sciences, Northern Illinois University, DeKalb, IL, USA 60115

^3^ SADABE Madagascar, Antananarivo, Madagascar

^4^ Columbus Zoo and Aquarium, Powell, OH, USA 43065

^5^ Mahaliana Labs, Lot II B 55 G Amboditsiry, Antananarivo 101, Madagascar

^6^ Department of Anthropology, University of Massachusetts Amherst, Amherst, MA, USA 01003

^7^ Department of Evolutionary Anthropology, Duke University, Durham, NC 27708

* corresponding author

**Supplementary Table S1:** Infant body masses recorded for *Propithecus diadema* at Tsinjoarivo, Madagascar.

| **Capture Date** | **Group** | **Estimated Age** | **Mass (g)** |
| --- | --- | --- | --- |
| 6 June 2015 | FRAG6 | 3 days (est.) | 143 |
| 22 July 2008 | CONT4 | 6-27 days (est. 7 days) | 145 |
| 18 July 2008 | CONT1 | 10 days (est.) | 205 |
| 18 July 2008 | CONT3 | 12 days (birth observed) | 170 |
| 29 July 2011 | CONT2 | 14 days (est.) | 270 |
| 27 July 2013 | CONT1 | 21 days (est.) | 234 |
| 23 July 2014 | FRAG7 | 4 weeks (est.) | 340 |
| 9 July 2008 | FRAG2 | 1 month (est.) | 265 |
| 10 July 2008 | FRAG4 | 1 month (est.) | 250 |
| 11 July 2008 | FRAG5 | 1 month (est.) | 202.5 |
| 24 July 2011 | CONT4 | 1 month (est.) | 180 |
| 19 July 2013 | FRAG6 | 1 month (est.) | 288 |
| 18 July 2013 | FRAG2 | 26-45 days (est. 33 days) | 275 |
| 20 July 2013 | FRAG4 | 39-45 days (est. 42 days) | 325 |
| 1 August 2014 | CONT2 | 1.5 months (est.) | 345 |
| 21 July 2013 | FRAG5 | >48 days (est. 50 days) | 347 |
| 10 November 2002 | FRAG1 | 5 month (est.) | 640 |

**Supplementary Table S2:** Definitions of morphometric measurements used during 188 captures of *Propithecus diadema* at Tsinjoarivo, Madagascar.

| **Measurement** | **Definition** |
| --- | --- |
| **Linear Somatic Measurements** | |
| Tail-Crown | Measured along dorsum from the level of the most superior point on the head (with the head in normal orthograde posture) to the tip of the last caudal vertebra |
| Tail | Measured along ventral side of the tail from junction of the base of the tail with the perianal area to tip of last caudal vertebra, with tail in line with rest of vertebral column |
| Hind limb | Measured from the groin to the end of pad of the longest digit, with the animal lying on its side and the hind limb extended vertically |
| Fibula | Measured from proximal to distal end of fibula as palpated on lateral surface of distal hind limb segment |
| Foot | Measured on the plantar surface from the heel to the end of the pad of the longest digit |
| Big Toe (Hallux) | Measured from the junction of the medial surface of the foot and big toe to the tip of pad of the big toe, with the big toe abducted (perpendicular to other digits) |
| Forelimb | Measured from the axilla to the tip of the pad of the longest digit, with the animal lying on its side and the arm extended vertically |
| Ulna | Measured from olecranon process to distal extremity of ulna, as palpated along posterior surface with antebrachium flexed (ulna perpendicular to humerus) |
| Hand | Measured on the palmar surface from the proximal edge of friction pad nearest the wrist to tip of the pad of the longest digit |
| Thumb (Pollex) | Measured from the junction between the lateral surface of the palm and the thumb to the tip of the thumb pad, with the pollex abducted (perpendicular to other digits) |
| **Circumferential Somatic Measurements** | |
| Chest Circumference | Circumference of chest at level of axilla, with arms raised |
| Biceps Circumference | Circumference of proximal forelimb segment at midpoint of humerus |
| Thigh Circumference | Circumference of proximal hind limb segment at midpoint of femur |
| **Testicular Measurements** | |
| Testicular Length | Maximum dimension of testis |
| Testicular Width | Maximum width of testis perpendicular to the maximum dimension |
